# Supplementary material for: Application of embedded soft PLC in the control system of rapier loom
Source: PLoS One. 2021 Sep 23;16(9):e0257629. doi: 10.1371/journal.pone.0257629 (PMC8460052; doi:10.1371/journal.pone.0257629)
Supplement: S1 File — (DOCX) [file pone.0257629.s001.docx]

Table 2 is a comparison table of actual warp tension and its error measured by genetic PID algorithm. A total of 100 sets of data are actually measured in the table. We randomly selects 10 sets of data and analyzes the selected data in Table 2.

**Table 2. The actual warp tension measured by genetic PID algorithm and its error comparison table**

| Number | Warp tension setting value （Kg） | Warp tension measured value （Kg） | Error（Kg） | Relative error（%） |
| --- | --- | --- | --- | --- |
| 1 | 150 | 149.01 | -0.99 | 0.66 |
| 2 | 150 | 147.25 | -2.75 | 1.83 |
| 3 | 150 | 148.17 | -1.83 | 1.22 |
| 4 | 150 | 150.67 | 0.67 | 0.45 |
| 5 | 150 | 154.49 | 4.49 | 2.99 |
| 6 | 150 | 156.08 | 6.08 | 4.05 |
| 7 | 150 | 155.73 | 5.73 | 3.82 |
| 8 | 150 | 151.89 | 1.89 | 1.26 |
| 9 | 150 | 149.57 | -0.43 | 0.29 |
| 10 | 150 | 145.21 | -4.79 | 3.19 |
| 11 | 150 | 147.55 | -2.45 | 1.63 |
| 12 | 150 | 149.93 | -0.07 | 0.05 |
| 13 | 150 | 152.47 | 2.47 | 1.65 |
| 14 | 150 | 157.28 | 7.28 | 4.85 |
| 15 | 150 | 154.35 | 4.35 | 2.90 |
| 16 | 150 | 150.19 | 0.19 | 0.13 |
| 17 | 150 | 147.72 | -2.28 | 1.52 |
| 18 | 150 | 145.11 | -4.89 | 3.26 |
| 19 | 150 | 148.36 | -1.64 | 1.09 |
| 20 | 150 | 149.95 | -0.05 | 0.03 |
| 21 | 150 | 151.27 | 1.27 | 0.85 |
| 22 | 150 | 153.16 | 3.16 | 2.11 |
| 23 | 150 | 150.85 | 0.85 | 0.57 |
| 24 | 150 | 149.13 | -0.87 | 0.58 |
| 25 | 150 | 146.25 | -3.75 | 2.50 |
| 26 | 150 | 148.37 | -1.63 | 1.07 |
| 27 | 150 | 151.02 | 1.02 | 0.68 |
| 28 | 150 | 153.61 | 3.61 | 2.41 |
| 29 | 150 | 155.93 | 5.93 | 3.95 |
| 30 | 150 | 155.08 | 5.08 | 3.39 |
| 31 | 150 | 152.96 | 2.96 | 1.97 |
| 32 | 150 | 150.57 | 0.57 | 0.38 |

| Number | Warp tension setting value （Kg） | Warp tension measured value （Kg） | Error（Kg） | Relative error  （%） |
| --- | --- | --- | --- | --- |
| 33 | 150 | 149.01 | -0.99 | 0.66 |
| 34 | 150 | 147.55 | -2.45 | 1.63 |
| 35 | 150 | 148.99 | -1.01 | 0.67 |
| 36 | 150 | 151.37 | 1.37 | 0.91 |
| 37 | 150 | 155.39 | 5.39 | 3.59 |
| 38 | 150 | 154.35 | 4.35 | 2.90 |
| 39 | 150 | 151.89 | 1.89 | 1.26 |
| 40 | 150 | 148.51 | -1.49 | 0.99 |
| 41 | 150 | 144.71 | -5.29 | 3.53 |
| 42 | 150 | 147.38 | -2.62 | 1.75 |
| 43 | 150 | 149.13 | -0.87 | 0.58 |
| 44 | 150 | 151.94 | 1.94 | 1.29 |
| 45 | 150 | 153.61 | 3.61 | 2.41 |
| 46 | 150 | 151.85 | 1.85 | 1.23 |
| 47 | 150 | 151.10 | 1.10 | 0.73 |
| 48 | 150 | 148.35 | -1.65 | 1.10 |
| 49 | 150 | 146.71 | -3.29 | 2.19 |
| 50 | 150 | 149.96 | -0.04 | 0.03 |
| 51 | 150 | 150.51 | 0.51 | 0.34 |
| 52 | 150 | 152.87 | 2.87 | 1.91 |
| 53 | 150 | 153.14 | 3.14 | 2.09 |
| 54 | 150 | 151.27 | 1.27 | 0.85 |
| 55 | 150 | 149.34 | -0.66 | 0.44 |
| 56 | 150 | 146.49 | -3.51 | 2.34 |
| 57 | 150 | 148.08 | -1.92 | 1.28 |
| 58 | 150 | 150.94 | 0.94 | 0.63 |
| 59 | 150 | 154.09 | 4.09 | 2.73 |
| 60 | 150 | 151.11 | 1.11 | 0.74 |
| 61 | 150 | 147.78 | -2.22 | 1.48 |
| 62 | 150 | 144.53 | -5.47 | 3.65 |
| 63 | 150 | 146.71 | -3.29 | 2.19 |
| 64 | 150 | 150.92 | 0.92 | 0.61 |
| 65 | 150 | 153.67 | 3.67 | 2.45 |
| 66 | 150 | 157.01 | 7.01 | 4.67 |
| 67 | 150 | 155.01 | 5.01 | 3.34 |

| Number | Warp tension setting value （Kg） | Warp tension measured value （Kg） | Error（Kg） | Relative error  （%） |
| --- | --- | --- | --- | --- |
| 68 | 150 | 152.23 | 2.23 | 1.49 |
| 69 | 150 | 149.65 | -0.35 | 0.23 |
| 70 | 150 | 145.53 | -4.47 | 2.98 |
| 71 | 150 | 142.69 | -7.31 | 4.87 |
| 72 | 150 | 146.43 | -3.57 | 2.38 |
| 73 | 150 | 149.17 | -0.83 | 0.55 |
| 74 | 150 | 151.32 | 1.32 | 0.88 |
| 75 | 150 | 156.06 | 6.06 | 4.04 |
| 76 | 150 | 152.76 | 2.76 | 1.84 |
| 77 | 150 | 150.61 | 0.61 | 0.41 |
| 78 | 150 | 148.46 | -1.54 | 1.03 |
| 79 | 150 | 145.94 | -4.06 | 2.71 |
| 80 | 150 | 149.31 | -0.69 | 0.46 |
| 81 | 150 | 153.53 | 3.53 | 2.35 |
| 82 | 150 | 157.42 | 7.42 | 4.95 |
| 83 | 150 | 154.59 | 4.59 | 3.06 |
| 84 | 150 | 150.81 | 0.81 | 0.54 |
| 85 | 150 | 147.00 | -3.00 | 2.00 |
| 86 | 150 | 145.51 | -4.49 | 2.99 |
| 87 | 150 | 149.93 | -0.07 | 0.05 |
| 88 | 150 | 152.04 | 2.04 | 1.36 |
| 89 | 150 | 150.66 | 0.66 | 0.44 |
| 90 | 150 | 147.37 | -2.63 | 1.75 |
| 91 | 150 | 149.82 | -0.18 | 0.12 |
| 92 | 150 | 151.17 | 1.17 | 0.78 |
| 93 | 150 | 154.23 | 4.23 | 2.82 |
| 94 | 150 | 150.07 | 0.07 | 0.05 |
| 95 | 150 | 147.73 | -2.27 | 1.51 |
| 96 | 150 | 150.91 | 0.91 | 0.61 |
| 97 | 150 | 152.15 | 2.15 | 1.43 |
| 98 | 150 | 148.67 | -1.33 | 0.89 |
| 99 | 150 | 151.09 | 1.09 | 0.71 |
| 100 | 150 | 149.86 | -0.14 | 0.09 |
