# Supplementary material for: Application of embedded soft PLC in the control system of rapier loom
Source: PLoS One. 2021 Sep 23;16(9):e0257629. doi: 10.1371/journal.pone.0257629 (PMC8460052; doi:10.1371/journal.pone.0257629)
Supplement: Attachment — Submitted filename: Response to Reviewers.docx [file pone.0257629.s002.docx]

Dear Editor and Reviewers:

I am very honored to receive your email sent to me on July 9, 2021. In response to your comments to me, I have carefully revised the documents to be submitted. But I am not sure how to submit my modified files correctly. However, as I explored the submission process, I once again submitted the files that I had not modified before. I first express my apologies to you for this. Then I want to ask you about an effective submission process.

Finally, thank you for your patience and I am waiting for your reply at any time.

On behalf of my co-authors, we are very grateful to you for giving us an opportunity to revise our manuscript. We appreciate you very much for your positive and constructive comments and suggestions on our manuscript entitled“Application of embedded soft PLC in the control system of rapier loom”(PONE-D-21-15942).

We have studied reviewers’ comments carefully and tried our best to revise our manuscript according to the comments. The following are the responses and revisions. I have made in response to the reviewers’ questions and suggestions on an item-by-item basis. Thank again to the hard work of the editor and reviewer!

**Response to the comments of academic editor：**

1. The manuscript was changed to meet the style requirements of PLOS ONE.
2. The reference list of the manuscript was checked and ensured that it was complete and correct.
3. The list of authors on the manuscript was revised and ensured that each author had contact with a certain institution.
4. Provided a revised funding statement and a statement about the role of funders in the research. Provides a statement about the authors’ contributions and clearly and accurately indicates the role of these authors in the research.
5. Provides an updated statement of competitive interest. And confirm that this business cooperation will not change compliance with all PLOS ONE policies on sharing data and materials.
6. Make sure to refer to figures 1, 2, 4, 6, 8, 9, 10, 11, 12, and 17 in the text and production will need this reference to link readers to the figures.

**Response to the comments of Reviewer #1：**

1. **Introduction section can be extended to add the issues in the context of the existing work.**

**Response:** In the introduction part of the thesis, the description of the related work of the existing embedded soft PLC system is expanded. And compared the advantages and disadvantages of existing related work and research.

2. **Literature review techniques have to be strengthened by including the issues in the current system and how the author proposes to overcome the same.**

**Response:** The literature review in the introduction section adds a comparison of the latest domestic and foreign related research. It highlights the necessity of the embedded soft PLC control system proposed in this paper in reducing the cost of maintenance and improving the programmability of the system. And a simple explanation of how this article solves the problems raised, that is, this article designs the hardware structure and software structure of the embedded soft PLC control system for specific problems. At the same time design the embedded soft PLC bottom drive system and the embedded soft PLC control system main program. Finally, using STM32 single-chip microcomputer as the main control unit and PLC programming method, effectively reducing the maintenance cost of the system later and effectively improving the programmability of the system.

3. **What is the motivation of the proposed work?**

**Response:** The proposed motive is to make the rapier loom better realize automated production, further improve the production efficiency of the rapier loom, increase the programmability of the system, and reduce the cost of system maintenance.

4. **Research gaps, objectives of the proposed work should be clearly justified.**

**Response:** Through the actual investigation of current rapier loom manufacturers at home and abroad, it is found that there are problems of high later maintenance cost and poor programmability for the rapier loom control system. The comparison of recent domestic and foreign related research shows that the related research literature based on embedded soft PLC has not solved the problems of high maintenance cost and poor system programmability that exist in the control system of rapier looms.

5. **The authors should consider more recent research done in the field of their study (especially in the years 2018 and 2020 onwards).**

**Response:** Added the latest domestic and foreign research around 2020 and compared and described its advantages and disadvantages.

6. **The paper needs to provide significant experimental details to correctly assess its contribution: What is the validation procedure used?**

**Response:** In the fourth part, the description of the key program analysis process in the embedded soft PLC system mentioned in the thesis is added.

7. **Kindly provide several references to substantiate the claim made in the abstract (that is, provide references to other groups who do or have done research in this area)**

**Response:** Added the latest domestic and foreign research around 2020 and compared and described its advantages and disadvantages. In the literature review in the introduction, some comparisons of the latest domestic and foreign related studies are added. It highlights the necessity of the embedded soft PLC control system proposed in this paper in reducing the cost of maintenance and improving the programmability of the system. Finally, this article adds a description of the advantages of the proposed system in the introduction. This article also refers to the two documents "A metaheuristic optimization approach for energy efficiency in the IoT networks" and "Green communication in IoT networks using a hybrid optimization algorithm". In this way, the origin of the design idea of the embedded soft PLC system proposed in this paper is described, and the rationality of the proposed method is proved.

8. **An error and statistical analysis of data should be performed.**

**Response:** By adding the traditional PID algorithm to the analysis of the error and the relative error of the warp tension measurement value. Compared with the PID control algorithm based on genetic algorithm adopted in this paper, the error and relative error of the loom control tension measured value are compared. It is found that the use of genetic algorithm to control the rapier loom in the embedded soft PLC control system has significantly reduced errors.This can show that the embedded soft PLC control system proposed in this paper can effectively improve the precision of loom tension control while reducing maintenance costs and improving system programmability, thereby effectively improving the production efficiency of the loom.

9. **The conclusion should state scope for future work**

**Response:** In the conclusion, the scope of future work is added to strengthen the research on the remote control system, simplify the system structure, and improve the degree of system intelligence.

10. **Discuss the future plans with respect to the research state of progress and its limitations.**

**Response:** This subject is not deep enough in the remote control system, simplifying the system structure, and improving the degree of system intelligence. Therefore, in the future, the improvement of the embedded soft PLC control system of the rapier loom is mainly reflected in the following aspects:

1. With the advancement of science and technology, in the future development, the research of remote control system is imperative. Therefore, in order to meet future development needs, wireless transmission modules or industrial Ethernet modules are added to the design of embedded soft PLC systems. While the transmission rate is increasing, it meets the needs of industrial interconnection.

2. The rapier loom system has many input and output channels. The design of this system meets the system requirements, but it increases the complexity of the system to a certain extent. In the future design of this subject, it is necessary to further develop new solutions to simplify the system structure on the basis of satisfying the system functions and make the whole system more compact.

3. In order to improve the degree of intelligence of the system, this subject should consider adding the functions of system fault diagnosis and intelligent prediction in the future design process.

**Response to the comments of Reviewer #2：**

**• In Introduction section, the drawbacks of each conventional technique should be described clearly.**

**Response:** In the introduction part, a more detailed description of the shortcomings of traditional rapier loom control technology. And through a more detailed comparison and description of the advantages and disadvantages of traditional technologies, it highlights the advantages and innovations of the embedded soft PLC control system studied in this paper.

**• Introduction needs to explain the main contributions of the work more clearly.**

**Response:** In the introduction, through describing the advantages and innovations of the embedded soft PLC control system compared with the previous rapier loom control system, the contribution of the research content of this article is highlighted. The contribution and innovation of this article lies in the development of a complete low-cost control system, that is, an embedded soft PLC control system. This system solves the problems of high maintenance cost and poor programmability in the rapier loom control system in the past. After that, based on this system platform, we completed a more effective control of the tension system of the loom through a PID algorithm optimized by genetic algorithm. Finally, we proved the advantages of the system we developed through the on-site debugging results, and verified the rationality and feasibility of the system.

**• The authors should emphasize the difference between other methods to clarify the position of this work further**

**Response:** By adding a description of the difference between the method proposed in this article and other methods in the last paragraph of the introduction. That is, compared with other control methods in the field of rapier looms, this system solves the problems of high maintenance cost and poor programmability that have occurred in the control system of rapier looms in the past.

**• The Wide ranges of applications need to be addressed in Introductions**

**Response:** A description of the scope of application of the control system proposed in the thesis is added in the last paragraph of the introduction. That is, the application range of the control system proposed in this article is very wide. The control system proposed in this paper is suitable for rapier loom control systems generally used in actual industrial production.

**• The objective of the research should be clearly defined in the last paragraph of the introduction section.**

**Response:** In the last paragraph of the introduction, the research goal of this article is pointed out. That is, the research goal of this article is to integrate the advantages of the single-chip control system and the PLC control system, reduce the maintenance cost of the system and improve the programmability of the system, thereby further improving the production efficiency of the rapier loom.

**• Add the advantages of the proposed system in one quoted line for justifying the proposed approach in the Introduction section. In literature survey under optimization the authors can refer A metaheuristic optimization approach for energy efficiency in the IoT networks. Green communication in IoT networks using a hybrid optimization algorithm**

**Response:** This article adds a description of the advantages of the proposed system in the introduction and draws reference to the two documents "A metaheuristic optimization approach for energy efficiency in the IoT networks" and "Green communication in IoT networks using a hybrid optimization algorithm". In this way, the origin of the design idea of the embedded soft PLC system proposed in this paper is described, and the rationality of the proposed method is proved.

**Changes to the reference list：Added citations and references to the following 4 papers in the article**

[13]Xu Xiao. Application of embedded soft PLC in coal mine control system[J]. Energy Technology and Management, 2020, 45(04):181-182.

[14] Gao Yanxiang. Design of embedded soft PLC control system for comprehensive excavation equipment [J]. Coal Mine Machinery, 2020, 41(07): 174-178.

[15]Shi Chunxiao. Intelligent controller design based on embedded soft PLC technology [J]. Computer Measurement and Control, 2020, 28(04): 126-130.

[16] Zhu Wei, Wang Hong, Li Shoubin, Zhao Wensheng. Design of roadheader control system based on embedded soft PLC [J]. Industry and Mine Automation, 2020, 46(02): 100-106.

We look forward to hearing from you regarding our submission. We would be glad to respond to any further questions and comments that you may have.
